# ESBL Production Among *E. coli* and *Klebsiella* spp. Causing Urinary Tract Infection: A Hospital Based Study

**DOI:** 10.2174/1874285801711010023

**Published:** 2017-04-28

**Authors:** Pooja Shakya, Dhiraj Shrestha, Elina Maharjan, Vijay K. Sharma, Rabin Paudyal

**Affiliations:** 1 Department of Microbiology, Kathmandu College of Science and Technology, Kathmandu, Nepal; 2 Department of Microbiology, Tri-Chandra Multiple College, Ghantaghar, Kathmandu, Nepal.; 3 Department of Biochemistry, Institute of Medicine, Tribhuvan University Teaching Hospital, Maharajgunj, Kathmandu, Nepal

**Keywords:** *E. coli*, Extended-spectrum β-lactamase, ESBL, *Klebsiella*, Nepal, Urinary tract infection, UTI

## Abstract

**Introduction:**

Increase in extended-spectrum β-lactamases (ESBL) producing microbes in recent years has led to limitations of treatment options. This study aimed to assess the prevalence of ESBL producing *E. coli* and *Klebsiella* spp. at a tertiary hospital in Nepal.

**Methods:**

A total of 2209 non-repetitive mid-stream urine (MSU) samples were collected during the study period (March to September 2014). Identification of the isolates was done by Gram's staining followed by biochemical tests. Antibiotic susceptibility testing was done by modified Kirby-Bauer disc diffusion method and interpretation was done following Clinical and Laboratory Standard Institute (CLSI) guidelines, 2013. ESBL screening among *E. coli* and *Klebsiella* spp. isolates were done using ceftriaxone, cefotaxime, ceftazidime and cefpodoxime. The confirmation was done by phenotypic disc diffusion test (combined disc method) using ceftazidime (30µg) and ceftazidime plus clavulanic acid (30/10µg), and cefotaxime (30µg) and cefotaxime plus clavulanic acid (30/10µg) disc as per CLSI guidelines.

**Results:**

A total of 451 samples showed significant bacteriuria with 365 (80.9%) *E. coli*, 17 (3.8%) *Klebsiella pneumoniae* and 3 (0.7%) *Klebsiella oxytoca*. Of 451 isolates, 236 (52.3%) were found MDR strains. By combined disk test, 33 (91.7%) *E. coli* and 3 (8.3%) *Klebsiella* spp. were found ESBL producers.

**Conclusion:**

Higher prevalence of ESBL producing *E. coli* and *Klebsiella* spp. was observed warranting prompt need of surveillance for effective management of such MDR strains.

## BACKGROUND

Urinary tract infection (UTI) is a spectrum of disease caused by microbial invasion of the genitourinary tract that extends from the renal cortex of the kidney to the urethral meatus [1]. UTI is an extremely common condition that occurs in both males and females of all ages. The prevalence and incidence of infection are higher in women than in men, which is likely the result of several clinical factors including anatomical differences, hormonal effects and behavior patterns [2]. The reported positive rate of UTI among Nepalese patients attending general hospitals ranged from 23.1% to 37.4% [3].

Bacteria are the major causative organisms and are responsible for more than 95% of UTI cases. *Escherichia coli* is the most prevalent causative organisms of UTI and is solely responsible for more than 80% of the infections [4].

Multiple drug resistance has significantly increased in recent years. There is a growing concern for multidrug-resistant Gram-negative bacteria which produce extended-spectrum β-lactamases (ESBLs) [5]. ESBLs are Class A β-lactamases that hydrolyse penicillin, oxyimino-cephalosporins, and monobactams but not cephamycins or carbapenems [6]. They are inhibited *in vitro* by clavulanate. ESBLs are often plasmid-mediated enzymes and have various genotypes. The most common are the SHV, TEM, and CTX-M types. Other clinically important types include VEB, PER, BEL-1, BES-1, SFO-1, TLA, and IBC [5]. ESBLs are primarily produced by the Enterobacteriaceae family of Gram-negative organisms, in particular *Klebsiella pneumoniae, K. oxytoca* and *E. coli* [7-9].They are also produced by other Gram-negative organisms, such as *Acinetobacter baumannii, Proteus* spp, *Pseudomonas aeruginosa* and *Salmonella* spp. [8, 10].

Since the first isolation of ESBL-producing organism in 1983 from Germany, it has been increasingly reported worldwide to date [11, 12].The prevalence of ESBL-positive isolates depends on a range of factors including species, geographic locality, hospital/ward, group of patients and type of infection, and large variations have been reported in different studies [13].

There are limited studies showing a high rate of ESBL in Nepal, where Enterobacteriaceae were found 28% to 67% [14]. The acquisition and expression of ESBLs enzymes among *Enterobactericeae* have posed a serious public health problem. Many parts of Nepal still lack the facilities for urine culture and antimicrobial susceptibility testing; this clearly leads to missing ESBL isolates. Delayed reporting of ESBL producing Gram-negative bacilli is associated with prolonged hospital stay, increased morbidity, increased motility and high health care costs [6]. Information on prevailing levels of antimicrobial resistance among common pathogens is useful in making an appropriate choice of empiric therapy [15]. This study would help clinicians to be aware of the potential of treatment failures associated with serious infections caused by these bacteria and guide for appropriate empirical antimicrobial therapy.

## MATERIALS AND METHODS

### Study Setting, Design and Population

This prospective cross-sectional study was done at Alka Hospital, Lalitpur (ISO 9001: 2008 standard) to assess the prevalence of ESBL production among the Gram-negative rods isolated from urine samples. All non-repetitive mid-stream urine (MSU) samples obtained during the study period (March to September 2014) were included in the study.

### Processing of Samples

All MSU samples were cultured on routine culture media by semi-quantitative method as described in World Health Organization (WHO) manual [16]. In short, 1μL of urine was inoculated on MacConkey and blood agar plate (HiMedia Laboratories Pvt. Ltd., India) by streaking using calibrated loop, and incubated aerobically for 18-48hrs at 37ᵒC. Growth of 100 colonies or more, *i.e.* 10^5^ colony forming units (CFU)/mL urine, was considered as culture positive. Isolation and identification of isolates were done following their morphology in Gram's staining, cultural characteristics and biochemical properties, as per the Manual of Clinical Microbiology [17].

### Antimicrobial Susceptibility Testing

Antibiotic susceptibility testing of all isolates was performed by Kirby-Bauer’s disc diffusion method and interpretation of the results was done as described in CLSI 2013 [18]. Antibiotic discs (HiMedia Laboratories Pvt. Ltd., India) used were amoxicillin (10μg), cefixime (10μg), cefotaxime (30μg), cefpodoxime (30μg), ceftazidime (30μg), ceftriaxone (30μg), ciprofloxacin (5μg), cotrimoxazole (1.25/23.75μg), gentamycin (10μg), imipenem (10μg), nitrofurantoin (300μg), norfloxacin (10μg), ofloxacin (5μg), piperacillin (100μg), and vancomycin (30μg). Control strains of *P. aeruginosa* ATCC 27853 and *E. coli* ATCC 25922 were used in parallel as a part of quality control. Organisms resistant to two or more classes of antimicrobial agents were considered to be multidrug resistance (MDR).

### Screening of ESBL-Producing Strains

According to CLSI guidelines, strains showing zone of inhibition of ≤25mm for ceftriaxone and/or ≤22mm for ceftazidime and/or ≤17mm for cefpodoxime and/or ≤27mm for cefotaxime were considered for conformational test for ESBL.

### Confirmation of ESBL-Producing Strains

ESBL production among potential ESBL-producing isolates was confirmed phenotypically using combined disc method. Comparison of the zone of inhibition was made for the ceftazidime (30µg) and cefotaxime (30µg) discs alone vs. that of the ceftazidime and cefotaxime discs containing clavulanic acid (10µg), when placed 25mm apart (center to center). Isolates showing an increase in zone diameter of ≥5 mm around either of the clavulanate combined discs compared to that of the disc alone was considered ESBL producer. *K. pneumoniae* ATCC 700603 was used as control strains.

### Ethical Consideration

Ethical approval was not required to carry out this work as the bacterial isolates were collected as part of routine patient care investigation in the hospital.

## RESULT

This study was conducted among patients suspected of UTI visiting Alka Hospital. A total of 2209 non-repetitive MSU samples were collected from patients for urine culture. Only 451 (20.4%) samples showed significant growth.

### Distribution of Samples among Patients

The ratio of number of inpatients to outpatients was 1:2.96. Similarly, number of male patients to female patients ratio was 1:2.2; females were higher in all cases. (Table **1**).

### Distribution of Bacterial Isolates

Of the 451 isolates, the most predominant isolate was *E. coli* 365 (80.9%). Among 451 isolates, 236 (52.3%) were MDR; *E. coli* with the most MDR share *i.e.* 188 (79.7%). Out of total, 168 (85.2%) isolates passed screening test for ESBL but only 36 (21.4%) of them were ESBL producers. *E. coli* was the highest ESBL producers *i.e.* 33 (91.7%) and followed by *K. pneumoniae i.e.* 3 (8.3%) (Table **2**).

### Antibiotics Resistivity Pattern of the Isolates

**Fig. (1):**
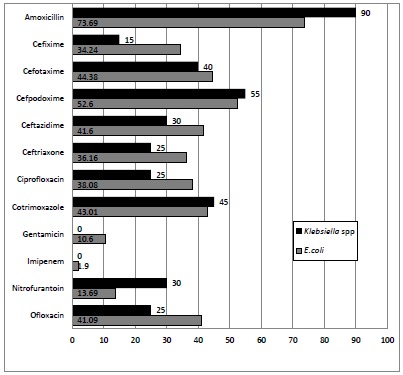
Antibiotics resistivity profile of isolates.

*E. coli* were highly resistant to amoxicillin 269 (73.69%) and least resistant to imipenem 7(1.9%). Likewise, most *Klebsiella* spp. were highly resistant to amoxicillin 18 (90%) and least resistant to gentamycin 0 (0%) and imipenem 0 (0%) (Fig. **1**).

### Distribution of ESBL Producing Isolates

The prevalence of ESBL producing *E. coli* were higher in female 27 (81.8%) compared to male 6 (18.2%). Similarly, the prevalence of ESBL producing *Klebsiella* spp. was 2 (66.7%) in female and 1(33.3%) in male (Table **3**).

The prevalence of ESBL production in *E. coli* was higher in 21-40 years age group (Table **4**).

## DISCUSSION

The emergence and rapid spread of multidrug resistant isolates are of great concern worldwide; among them, ESBL producing Enterobacteriaceae has been major concern. During the past decades, ESBLs producing Gram-negative bacilli especially *E. coli and K. pneumoniae* have emerged as serious pathogens both in hospital and community acquired infections worldwide. The study was conducted among the patient suspected of UTI visiting Alka Hospital, Lalitpur, Nepal. A total of 2209 non-repetitive MSU samples were included during the study period (March to September 2014). Of these samples, only 451 (20.4%) showed significant growth. Higher growth rates were reported in similar studies of 26.6% [19], 30.8% [20], 41.7% [21], but lower growth rates were reported in other similar studies of 9% [22], 17.4% [23], 19.7% [24].

In this study, the ratio of number of inpatients to outpatients was 1:2.96. And among positive cases, the ratio was 1:4.01. This signifies more prevalence of UTI in community. In this study, the number of male patients to female patients ratio was 1:2.2. And among positive cases, the ratio was 1:3.21. Females were higher in both cases which was involuntary recruitment bias. Females are more frequently affected by(particularly cystitis) due to colonization of urethra with colonic Gram-negative bacteria because of its proximity to anus and short length of urethra [25]. The patient’s sex is risk factor of UTI [26].

*E. coli* (80.9%) was the most common pathogens isolated. *E. coli,* including other enterobacteria, are likely to have caused infection after colonization of the periurethral area by gastrointestinal tract flora [27]. This accords with other studies [21, 22, 24, 28-34]. However, discords with following studies [35-38].

Antimicrobial resistance is now accepted as a major problem in public health and patient care. It is more troublesome to developing countries. In this study, *E. coli* were highly resistant to amoxicillin 269 (73.7%) and least resistant to imipenem 7 (1.9%). This is similar to reports of different studies [21, 23, 24, 29-31, 33, 35-41]. Likewise, most *Klebsiella* spp. were highly resistant to amoxicillin 18 (90%) and least resistant to gentamycin 0 (0%) and imipenem 0 (0%). Similar result was shown in different studies [29, 33, 35, 36, 38, 41, 42]. Imipenem was the most effective drug, however, it should not be administered as empirical drug unless infection is life threatening, as carbapenems are considered the drug of last resort. In case, gentamycin or nitrofurantoin could be the empiric choice. In this study, 236 (52.3%) isolates were MDR. The result accords with other studies showing 55.9% [43], 64% [23], and 64.9% [24]of MDR isolates.

Identifying ESBL producing organisms is a major challenge in clinical settings and, due to the selective pressure caused by heavy use of expanded-spectrum cephalosporins, lapses in effective infection control measures and affinity of these enzymes for different substrates, outbreaks are increasing [44]. The prevalence of ESBL producing Enterobacteriaceae varies greatly among country and among the hospitals within the country. Less than 1% to greater than 70% ESBLs producers are reported worldwide. Of 385 isolates of *E. coli* and *Klebsiella* spp. 168 isolates passed ESBL screening test but only 36 (9.4%) were confirmed ESBL producers phenotypically. The prevalence of ESBL production was higher in 21 - 40 years age group. The prevalence was higher in this age group as most isolates, accounting 41%, were isolated from this group. Besides, self medication practice which is high in this age group, could have further accounted for higher prevalence [45, 46]. A higher prevalence of ESBL production was observed in *E. coli* followed by *K. pneumoniae*. The findings are in agreement with the study [11], however, contrary to the findings of other study [47] of 365 *E. coli* isolates, 33 (9.0%) were ESBL producers. This accords with the other studies [33, 35, 41, 48], however, discords with following studies [22, 24, 29-32, 36-38]. Likewise, of 20 *Klebsiella* spp. isolates, 3 (15%) were ESBL producers. This accords with other studies [22, 29, 35, 48], however, discords with following studies [32, 33, 36, 41].

ESBL-producing strains are creating significant therapeutic problems since these pathogens are resistant to a wide range of β-lactams, including third generation cephalosporins as well as have potential for plasmid mediated quinolone and carbapenem resistance. Antibacterial choice is often complicated by MDR leading to over-prescription of antibiotics. As indicated by the present finding together with previous findings, it appears to be mandatory to include ESBL detection in routine laboratory practice so as to limit the rapid spread of ESBL-producing organisms.

## CONCLUSION

In this study, *E. coli* was found to be the most predominant MDR isolate. The prevalence of ESBL producing *E. coli* and *Klebsiella* spp. was higher. The majority of ESBL producing *E. coli* and *Klebsiella* spp. were resistant to the in-use antibiotics used for treatment of UTI. Imipenem was the most effective antibiotic and could be the drug of choice for treatment of infections caused by ESBL strains. This clinical threat of increased ESBL prevalence is creating significant therapeutic problems prompting an immediate need to formulate strategic policy initiatives to reduce their prevalence.

## Figures and Tables

**Table 1:** Distribution of samples among inpatients and outpatients.

**Urine Culture**	**Inpatients**		**Outpatients**	
	**Male (%)**	**Female (%)**	**Male (%)**	**Female (%)**
Significant growth	21(15.2)	69(16.4)	86(15.6)	275(25.0)
Non-significant growth	117(84.8)	351(83.6)	466(84.4)	824(75.0)
**Total**	138(100)	420(100)	552(100)	1099(100)

**Table 2:** Distribution of isolates.

**Organisms**	**Total isolates**	**No. of MDR strains**	**ESBL screening positive**	**ESBL confirmed **
*E. coli*	365(80.9%)	188(79.7%)	160	33 (91.7%)
*K. oxytoca*	3 (0.7%)	1(0.4%)	1	0 (0%)
*K. pneumoniae*	17(3.8%)	8(3.4%)	7	3 (8.3%)
*Acinetobacter* spp	1 (0.2%)	0 (0%)	NT	NT
*Citrobacter* spp	4 (0.9%)	1 (0.4%)	NT	NT
*Enterobacter* spp	3 (0.7%)	1 (0.4%)	NT	NT
*Enterococcus* spp	3 (0.7%)	2 (0.8%)	NT	NT
*Morganella* spp	3 (0.7%)	1 (0.4%)	NT	**NT**
*Proteus mirabilis*	12 (2.7%)	7 (3%)	NT	NT
*Proteus vulgaris*	7 (1.6%)	6 (2.5%)	NT	NT
*Providencia* spp	22 (4.9%)	12 (5.1%)	NT	NT
*Pseudomonas* spp	7 (1.6%)	7 (3%)	NT	NT
*Staphylococcus* spp	4 (0.9%)	2 (0.8%)	NT	NT
**Total**	**451 (100%)**	**236 (100%)**	**168**	**36 (100%)**

NT=not tested

**Table 3:** Sex wise distribution of ESBL producing isolates.

**Sex**	***E. coli***				***Klebsiella *spp.**			
	***E. coli *isolates(%)**	**MDR(%)**	**Screening positive(%)**	**ESBL Producers(%)**	***Klebsiella *spp. isolates(%)**	**MDR(%)**	**Screening positive(%)**	**ESBL Producers(%)**
Male	77 (21.1)	43 (22.9)	37 (23.1)	6 (18.2)	4 (20)	3 (33.3)	3 (37.5)	1 (33.3)
Female	288 (78.9)	145 (77.1)	123 (76.9)	27 (81.8)	16 (80)	6 (66.7)	5 (62.5)	2 (66.7)
**Total**	**365 (100)**	**188 (100)**	**160 (100)**	**33 (100)**	**20 (100)**	**9 (100)**	**8 (100)**	**3 (100)**

**Table 4:** Age wise distribution of ESBL Producing isolates.

**Age group (years)**	***E. coli***				***Klebsiella* spp.**			
	***E. coli *isolates(%)**	**MDR(%)**	**Screening positive(%)**	**ESBL producer(%)**	***Klebsiella *spp. isolates(%)**	**MDR(%)**	**Screening positive(%)**	**ESBL producer(%)**
<=10	31 (8.5)	16 (8.5)	12 (7.5)	1 (3)	4 (20)	2 (22.2)	2 (25)	0 (0)
11-20	16 (4.38)	8 (4.3)	5 (3.13)	1 (3)	2 (10)	1 (11.1)	2 (25)	1 (33.3)
21-30	95 (26)	44 (23.4)	31 (19.4)	9 (27.3)	2 (10)	1 (11.1)	1 (12.5)	0 (0)
31-40	59 (16.2)	25 (13.3)	25 (15.6)	8 (24.2)	2 (10)	1 (11.1)	1 (12.5)	1 (33.3)
41-50	44 (12.1)	27 (14.4)	23 (14.4)	3 (9.1)	0 (0)	0 (0)	0 (0)	0 (0)
51-60	37 (10.1)	15 (8)	19 (11.9)	6 (18.2)	3 (15)	1 (11.1)	0 (0)	0 (0)
61-70	45 (12.3)	30 (16)	24 (15)	3 (9.1)	5 (25)	2 (22.2)	2 (25)	1 (33.3)
70+	38 (10.4)	23 (12.2)	20 (12.5)	1 (3)	2 (10)	1 (11.1)	0 (0)	0 (0)
**Total**	**365 (100)**	**188 (100)**	**160 (100)**	**33 (100)**	**20 (100)**	**9 (100)**	**8 (100)**	**3 (100)**
